# Finite-time and predefined-time dynamical systems for solving strongly pseudomonotone mixed equilibrium problems

**DOI:** 10.1371/journal.pone.0357046

**Published:** 2026-09-01

**Authors:** Qian Wen, Vajahat Karim Khan, Qing-Bo Cai, Md. Kalimuddin Ahmad

**Affiliations:** 1 Fujian Key Laboratory of Big Data Application and Intellectualization for Tea Industry, College of Mathematics and Computer, Wuyi University, Nanping, Fujian, China; 2 Department of Mathematics, Faculty of Science, Aligarh Muslim University, Aligarh, Uttar Pradesh, India; 3 Fujian Provincial Key Laboratory of Data-Intensive Computing, Key Laboratory of Intelligent Computing and Information Processing, School of Mathematics and Computer Science, Quanzhou Normal University, Quanzhou, Fujian, China; Aalto University, FINLAND

## Abstract

In this paper, we propose a class of proximal dynamical systems (PDSs) with finite-time (FT) stability and predefined-time (PdT) stability for solving strongly pseudomonotone mixed equilibrium problems (MEPs) in Hilbert spaces. Unlike existing approaches for variational inequalities and standard equilibrium problems, the proposed framework is developed specifically for MEPs and provides two distinct dynamical models with FT and PdT convergence. Under the assumptions of strong pseudomonotonicity and Lipschitz-type continuity, we establish the existence and uniqueness of the equilibrium solution, prove the global exponential stability of the associated continuous-time nominal system, and show that its discrete-time discretization yields a proximal-type algorithm with linear convergence. Furthermore, we construct an FT stable PDS whose equilibrium point coincides with the solution of the MEP and derive sufficient conditions for finite-time convergence. In addition, we introduce a novel PdT dynamical system that guarantees convergence within a prescribed time independent of the initial conditions. Numerical experiments are provided to demonstrate the effectiveness of the proposed methods, indicating that the PdT system achieves faster convergence compared to the nominal and FT systems. Finally, an application to sparse signal recovery in compressed sensing demonstrates the practical effectiveness of the proposed PdT scheme.

## 1. Introduction

Let ${\mathbb{R}}^{d}$ denote the real *d*-dimensional Euclidean space equipped with the inner product ⟨·,·⟩ and the induced norm ‖·‖. The equilibrium problem (EP) is a general framework that includes many well-known problems as special cases. Examples include optimization problems, variational inequalities, saddle point problems, Nash equilibria in noncooperative games, and fixed point problems; see [1–7]. The importance of EPs lies in their ability to unify these diverse models within a single mathematical framework. Due to their wide applicability, equilibrium problems have attracted considerable attention in recent years, and numerous applications have been successfully modeled and analyzed using equilibrium formulations. For a comprehensive discussion of the existence of equilibrium solutions and their solution approaches, we refer the reader to [8].

Given that EPs constitute a unified modeling framework, many solution techniques presented for specific problems can be naturally extended to this setting; see, for instance, [8–14]. Among these approaches, fixed–point–type methods play a central role due to their simple structure and practical effectiveness. These methods are closely related to proximal point and projection techniques originally developed for variational inequalities (VIs) [1, 2, 13, 15, 16]. Previous work [17] has shown that fixed-point techniques are effective for solving strongly monotone equilibrium problems, and their convergence has been proven to be linear [18].

In recent years, continuous-time dynamical systems have attracted significant attention for solving variational inequalities, fixed-point problems, and inclusion problems [19–24]. In particular, the fixed-time convergent proximal dynamical system (PDS) for mixed variational inequalities (MVIs) proposed in [25] was established under the assumptions of Lipschitz continuity and strong monotonicity; see also [26, 27]. More recently, predefined-time (PdT) convergence has gained considerable interest because it guarantees convergence within a prescribed time independent of the initial conditions. Its effectiveness has been demonstrated in optimization, distributed decision-making, sparse signal recovery, quadratic programming, synchronous control, and variational inequality problems [28–33].

From this perspective, several dynamical and neurodynamic approaches have been introduced for equilibrium and variational inequality problems, including PdT and finite-time (FT) proximal dynamics, projection neural networks, and adaptive step-size schemes for (pseudo-)monotone and mixed models [11, 34–38]. Nevertheless, most existing studies on PdT convergence focus on equilibrium problems (EPs). To the best of our knowledge, FT and PdT proximal dynamical approaches for mixed equilibrium problems (MEPs) remain largely unexplored. In many practical applications, equilibrium-based models must be solved under strict time constraints, where asymptotic convergence may be insufficient. In particular, MEPs naturally arise in network resource allocation, traffic equilibrium, distributed optimization, game theory, and sparse signal recovery, where the equilibrium structure combines both operator interactions and nonsmooth regularization effects [20, 34].

The mixed structure of MEPs, involving the variational term Ψ(u)−Ψ(w) together with the operator term ⟨Υ(w),u−w⟩, enables the modeling of constrained, nonsmooth, and multi-agent decision processes within a unified framework. In such applications, guaranteeing convergence within finite or predefined time is particularly important for real-time optimization, fast decision-making, and online control tasks [30, 31]. FT convergence ensures that the system trajectories reach the equilibrium state within a bounded settling time depending on the initial conditions, whereas PdT convergence guarantees convergence within a user-prescribed time bound independent of the initial state. Compared with asymptotic or exponential convergence, PdT convergence provides stronger predictability and robustness, which are highly desirable in engineering applications requiring guaranteed response speed [39, 40]. Although FT and PdT dynamical approaches have been extensively studied for variational inequalities and standard equilibrium problems [25, 27, 36, 38, 41], their extension to strongly pseudomonotone MEPs remains largely unexplored due to the additional analytical difficulties introduced by the nonsmooth mixed structure and proximal operator formulation.

Motivated by this research gap, this paper studies proximal dynamical systems (PDSs) for solving strongly pseudomonotone MEPs in Hilbert spaces. The proposed framework combines fixed-point reformulations, proximal operator techniques, and Lyapunov-based stability analysis to develop dynamical models with provable stability and convergence properties. In particular, we develop novel PDSs that incorporate FT and PdT stability analysis with convergence mechanisms.

The main contributions of this paper are summarized as follows:

1. **First**, we propose a novel first-order proximal dynamical system (PDS) for solving strongly pseudomonotone MEPs. Under strong pseudomonotonicity and Lipschitz-type continuity assumptions, the introduced system admits a unique equilibrium solution and achieves global exponential stability. Furthermore, a discrete-time realization obtained via finite-difference discretization leads to a proximal-type algorithm with linear convergence, thereby extending existing dynamical approaches beyond standard equilibrium and VI problems.
2. **Second**, unlike the existing FT PDSs developed mainly for VIs and standard EPs [21, 25, 27, 36], we propose a new FT proximal dynamical framework for strongly pseudomonotone MEPs. By combining proximal operator techniques with Lyapunov-based stability analysis, sufficient conditions guaranteeing FT convergence are derived.
3. **Third**, we propose a novel PdT PDS for strongly pseudomonotone MEPs. In contrast to existing PdT dynamical approaches [30, 31, 38, 41], the proposed framework incorporates the mixed equilibrium structure together with nonsmooth proximal mappings while guaranteeing convergence within a user-prescribed time independent of the initial conditions and discretization step size. To the best of our knowledge, such a PdT proximal dynamical framework for strongly pseudomonotone MEPs has not been previously investigated.
4. Numerical experiments are presented to demonstrate the effectiveness of the proposed methods. The results illustrate the convergence behavior of the presented PdT PDS and provide comparisons with the nominal and FT PDS.
5. Finally, an application to sparse signal recovery in compressed sensing is presented to demonstrate the practical effectiveness of the proposed PdT scheme.

The remainder of this paper is organized as follows. In Section 2, we present some preliminary concepts, assumptions, and auxiliary results that will be used throughout the paper. Section 3 introduces a nominal proximal dynamical system for solving strongly pseudomonotone MEPs and establishes its existence, uniqueness, and exponential stability properties. In Section 4, a FT stable PDS is proposed and its convergence analysis is presented. Section 5 develops a PdT PDS and establishes its PdT stability. Numerical experiments together with an application to sparse signal recovery are presented in Section 6 to illustrate the effectiveness of the proposed methods. Finally, concluding remarks and future research directions are provided in Section 7.

## 2. Preliminary results with notations

In this section, we present the construction of the proposed PDS together with the related definitions and auxiliary results.

Consider the general dynamical system


$$\begin{array}{c} \frac{dw}{dt}=𝒢(w(t)),\;\text{with}\;w(0)=w_{0}\in {\mathbb{R}}^{d}, \end{array}$$ (1)

where $𝒢:𝒞\to {\mathbb{R}}^{d}$ is a continuous function defined on an open set $𝒞\subseteq {\mathbb{R}}^{d}$ containing the origin, with 𝒢(0)=0.

**Definition 1.** *[21, 27] A point* ${𝔴}^{*}\in {\mathbb{R}}^{d}$ *is an equilibrium point of* (1) *if* $𝒢({𝔴}^{*})=0.$ *An equilibrium point* ${𝔴}^{*}$ *is said to be*

*(a)* ***Lyapunov stability*** *if, for any* ε>0*,* ∃ δ>0 *such that (s. t.)*


$$\begin{array}{c} \|w_{0}-{𝔴}^{*}\|<\delta ⟹\forall t\geq t_{0},\;\|w(t)-{𝔴}^{*}\|<\varepsilon . \end{array}$$

*(b)* ***FT convergence*** *if* ∃ *a FT,* $𝒯:{\mathbb{R}}^{d}⧵\{0\}\to (0,\infty )\;(\text{i.e. }𝒯<\infty )$ *s. t. for all* w(0)∈N⧵{0},


$$\begin{array}{c} \lim_{t\to 𝒯}w(t)=0\;\text{and}\;𝒯=𝒯(w(0))<\infty . \end{array}$$

*(c)* ***Asymptotically stable*** *if* ∃ δ>0 *s. t. all solutions starting in* $B({𝔴}^{*},\delta )$ *satisfy*


$$\begin{array}{c} \lim_{t\to +\infty }w(t)={𝔴}^{*}. \end{array}$$

*(d) If* ∃, δ>0 *and constant* η>0 *and* ϱ>0 *s. t.*


$$\begin{array}{c} \|w(t)-{𝔴}^{*}\|\leq \eta \|w(0)-{𝔴}^{*}\|e^{-\varrho t},\;\forall t\geq 0. \end{array}$$

*Then we say that* ${𝔴}^{*}$ *is* ***exponentially stable*** *for all initial conditions* $w(0)\in B({𝔴}^{*},\delta )$.

**Remark 1.** *A solution w(t) of system (*1*) exists on an interval containing t*_*0*_ *= 0 if* 𝒢 *is continuous on its domain. Additionally, the uniqueness of the solution to (1) follows from the local Lipschitz continuity of* 𝒢*. Any equilibrium point of (1) that is not at the origin can be translated to the origin by a suitable change of variables (see* [42]*). In particular, let* $\bar{w}$ *be a nonzero equilibrium point of (1) and define* $v:=w-\bar{w}$*. Then, in terms of the new variable u,*


$$\begin{array}{c} \dot{v}=\dot{w}=ℱ(v),\;v(0)=v_{0}\in {\mathbb{R}}^{d}, \end{array}$$

*the origin* 𝔲=0 *becomes an equilibrium point, where* $ℱ(v):=𝒢(v+\bar{w})$*. Hence, Definition (1) applies whether or not the system* $\dot{w}=𝒢(w)$ *has its equilibrium point at the origin.*

**Definition 2.** *[*40*] For a preassigned time* ${𝒯}_{c}>0$*, which is independent of initial conditions and system parameters, the system* (1) *is said to be PdT convergent within* ${𝒯}_{c}$ *if*


$$\begin{array}{c} \lim_{t\to {𝒯}_{c}}\|w(t)\|=0,\;w(t)=0,\;\forall t\geq {𝒯}_{c}. \end{array}$$

**Lemma 1** (Pythagoras identity). *For any* $a,b,c\in {\mathbb{R}}^{d}$*, it holds that* $2\langle a-b,c-b\rangle =\|a-b{\|}^{2}+\|c-b{\|}^{2}-\|a-c{\|}^{2}$*.*

### 2.1. Mixed equilibrium problem

Suppose $\Omega \text{≠}\emptyset \subseteq {\mathbb{R}}^{d}$, which is closed and convex. Consider a bifunction Γ:Ω×Ω→ℝ s. t. Γ(w,w)=0 ∀ w∈Ω, and Γ(w,·) is convex, lower semicontinuous, and subdifferentiable on Ω. The equilibrium problem in the sense of [4] is to find ${𝔴}^{*}\in \Omega$ s. t.


$$\begin{array}{c} \Gamma ({𝔴}^{*},𝔲)\geq 0\;\text{for all }𝔲\in \Omega . \end{array}$$

When we take $\Psi :{\mathbb{R}}^{d}\to \mathbb{R}\cup \{+\infty \}$ is a proper, l.s.c and convex real-valued function, and $\Upsilon :{\mathbb{R}}^{d}\to {\mathbb{R}}^{d}$ is a vector-valued function. Then, we take the mixed equilibrium problem (MEP) [34] as follows:


$$\begin{array}{c} \Gamma (w,𝔲)=\langle \Upsilon (w),𝔲-w\rangle +\Psi (𝔲)-\Psi (w)\;\forall w,𝔲\in {\mathbb{R}}^{d}, \end{array}$$ (2)

In this context, the MEPs associated with Γ is denoted by MEP(Γ,Ω), which is equivalent to solving the MVI (Υ,Ω) problem. The MVI problem seeks a point ${𝔴}^{*}\in \Omega$ that satisfies the condition


$$\begin{array}{c} \langle \Upsilon ({𝔴}^{*}),𝔲-{𝔴}^{*}\rangle +\Psi (𝔲)-\Psi ({𝔴}^{*})\geq 0,\;\forall 𝔲\in {\mathbb{R}}^{d}, \end{array}$$

Therefore, set of solutions of MEP(Γ,Ω) is denoted by Sol(Γ,Ω). Thus, for any ${𝔴}^{*}\in \mathrm{Sol}(\Gamma ,\Omega )$, then from (2), we get $\Gamma ({𝔴}^{*},𝔲)\geq 0$. The monotonicity of the bifunction Γ defined above is directly related to the well-established concept of (generalized) monotonicity of the mapping Υ (see [34, 43]). For every μ>0, define the proximal operator associated with Ψ by ${\mathrm{prox}}_{\mu \Psi }(v)$ as:


$$\begin{array}{c} {\mathrm{prox}}_{\mu \Psi }(v)=\text{arg}\min_{z\in \Omega }\left\{\Psi (z)+\frac{1}{2\mu }\|v-z{\|}^{2}\right\}\;\forall v\in \Omega . \end{array}$$ (3)

The operator ${\mathrm{prox}}_{\mu \Psi }$ plays a crucial role in the dynamic system by incorporating the proximity term, which guides the solution towards the MEPs in a manner that respects the geometry of the set Ω.

**Remark 2** *Another notable special case of an MEP is the saddle point problem. Let* $\Omega_{1}\subset H_{1}$ *and* $\Omega_{2}\subset H_{2}$*, where H*_*1*_ *and H*_*2*_ *are real Hilbert spaces. A point* ${𝔴}^{*}=(w_{1}^{*},w_{2}^{*})\in \Omega_{1}\times \Omega_{2}$ *is called a saddle point of the function* $𝔈:\Omega_{1}\times \Omega_{2}\to \mathbb{R}$ *if*


$$\begin{array}{c} 𝔈(w_{1}^{*},{𝔲}_{2})\leq 𝔈(w_{1}^{*},w_{2}^{*})\leq 𝔈({𝔲}_{1},w_{2}^{*})\;\text{for all }({𝔲}_{1},{𝔲}_{2})\in \Omega_{1}\times \Omega_{2}. \end{array}$$

*A saddle point of* 𝔈 *can be characterized as a solution of the* MEP(Γ,Ω)*, where* $\Omega =\Omega_{1}\times \Omega_{2}$*, and*


$$\begin{array}{c} \Gamma \{(w_{1},w_{2}),({𝔲}_{1},{𝔲}_{2})\}=𝔈({𝔲}_{1},w_{2})-𝔈(w_{1},{𝔲}_{2}). \end{array}$$

*In fact, a saddle point of* 𝔈 *is precisely a Nash equilibrium in a two-player zero-sum game, with cost functions* 𝔈 *and* −𝔈 *for the two players, respectively.*

Now, we introduce the definitions, assumptions, and lemmas required throughout the paper. A bifunction Γ:Ω×Ω→ℝ is said to satisfy the following conditions:

1. Γ is **strongly monotone** with modulus θ>0 on Ω if
  $$\begin{array}{c} \Gamma (w,𝔲)+\Gamma (𝔲,w)\;\leq \;-\theta \|w-𝔲{\|}^{2}\;\forall w,𝔲\in \Omega . \end{array}$$
2. Γ is **monotone** on Ω, if
  $$\begin{array}{c} \Gamma (w,𝔲)+\Gamma (𝔲,w)\;\leq \;0\;\forall w,𝔲\in \Omega . \end{array}$$

**Assumption 1.** Γ *is* ***strongly pseudomonotone*** *with modulus* ζ>0 *on* Ω*, if*


$$\begin{array}{c} \Gamma (w,𝔲)\geq 0\;\Rightarrow \;\Gamma (𝔲,w)\leq -\zeta \|w-𝔲{\|}^{2}\;\forall w,𝔲\in \Omega . \end{array}$$

**Assumption 2.** Γ *is* ***pseudomonotone*** *on* Ω*, if*


$$\begin{array}{c} \Gamma (w,𝔲)\geq 0\;\Rightarrow \;\Gamma (𝔲,w)\leq 0\;\forall w,𝔲\in \Omega . \end{array}$$

**Assumption 3.** Γ *is a* ***Lipschitz-type condition*** *on* Ω *if* ∃ *a constant L > 0 s. t.*


$$\begin{array}{c} \Gamma (w,𝔲)+\Gamma (𝔲,z)\;\geq \;\Gamma (w,z)-L\|w-𝔲\|\;\|𝔲-z\|\;\forall w,𝔲,z\in \Omega . \end{array}$$

**Assumption 4.** $\mu L^{2}<2\zeta$.

**Remark 3.** *The implications* (1)⇒(2)*,* (1)⇒(Assumption 1)*,* (Assumption 1)⇒(Assumption 2)*, and* (2)⇒(Assumption 2) *are immediate. Moreover, under Assumption 1, the solution of* MEP(Γ,Ω) *is unique whenever it exists. Indeed, let* $w^{*},u^{*}\in Sol(\Gamma ,\Omega )$*. Then* $\Gamma ({𝔲}^{*},{𝔴}^{*})\geq 0$ *and by strong pseudomonotonicity* $-\Gamma ({𝔲}^{*},{𝔴}^{*})\geq \zeta \|{𝔲}^{*}-{𝔴}^{*}{\|}^{2}.$ *Adding them gives* $0\geq \zeta \|{𝔲}^{*}-{𝔴}^{*}{\|}^{2},$ *hence* ${𝔴}^{*}={𝔲}^{*}$.

**Remark 4.** *Assumption 3, introduced by Quoc and Muu [10], is weaker than the Lipschitz-type condition formulated by Antipin [2]. The latter may be written in the following form:*


$$\begin{array}{c} |\;\Gamma (w,𝔲)-\Gamma (w,z)+\Gamma (a,𝔲)-\Gamma (a,z)\;|\;\leq \;L\;\|w-a\|\;\|𝔲-z\|,\;\forall \;a,w,𝔲,z\in \Omega . \end{array}$$ (4)

*Indeed, taking a = z in (4), we obtain Assumption 3. Moreover, Assumption 3 entails a Lipschitz-type condition as introduced by Mastroeni* [17]:


$$\begin{array}{c} \Gamma (w,𝔲)+\Gamma (𝔲,z)\;\geq \;\Gamma (w,z)-\alpha_{1}\|w-𝔲{\|}^{2}-\alpha_{2}\|𝔲-z{\|}^{2},\;\forall w,𝔲,z\in \Omega , \end{array}$$

*where* $\alpha_{1},\alpha_{2}>0$ *are given constants. If* $\Gamma (w,𝔲)=\langle \Upsilon (w),\;𝔲-w\rangle +\Psi (𝔲)-\Psi (w),\;\forall w,𝔲\in {\mathbb{R}}^{d},$ *i.e., if* MEP(Γ,Ω) *reduces to a MVIP, then* Γ *satisfies Assumption 3 whenever* Γ *is Lipschitz continuous with L > 0.*

**Lemma 2.** [44] *Let* μ>0*,* $\Psi :{\mathbb{R}}^{d}\to (-\infty ,+\infty ]$ *be a l.s.c. function, which is proper and convex. We denote by* ${\mathrm{prox}}_{\mu \Psi }$ *the proximal operator defined in (3). Then the following assertion is true.:*

*1. For any* $w\in {\mathbb{R}}^{d}$*, we have*


$$\begin{array}{c} \nu ={\mathrm{prox}}_{\mu \Psi }(w)\Leftrightarrow \langle \nu -w,a-\nu \rangle \geq \mu \Psi (\nu )-\mu \Psi (a),\;\forall a\in {\mathbb{R}}^{d}. \end{array}$$

2. $\left\|{\mathrm{prox}}_{\mu \Psi }(w)-{\mathrm{prox}}_{\mu \Psi }(𝔲)\|\leq \|w-𝔲\right\|$, $\forall w,𝔲\in {\mathbb{R}}^{d}$.

**Lemma 3.** *[30] Suppose* ∃ *a continuous, positive definite, and radially unbounded function* $𝔙:{\mathbb{R}}^{d}\to {\mathbb{R}}_{\geq 0}$ *s. t.*


$$\begin{array}{c} \dot{𝔙}(w(t))\leq -\frac{1}{\lambda p{𝒯}_{c}}\exp\left(\lambda 𝔙(w(t){)}^{p}\right)𝔙(w(t){)}^{q}, \end{array}$$

*where* ${𝒯}_{c}>0$, λ>0*, p > 0, q < 1, and* $\forall \;w(t)\in {\mathbb{R}}^{d}⧵\{0\}$*. Then, the equilibrium point of system* (1) *is PdT stable with* ${𝒯}_{c}$.

**Lemma 4.** *[36, 37]* *Assume that the Assumptions 1, 3, and 4 hold, and* ${𝔴}^{*}$ *is a solution to MEP* (2). *Let* $ℋ(w):={\mathrm{prox}}_{\mu \Psi }(w-\mu \Upsilon (w))$*,* (1−Ξ)∈(0,1) *and* $\Xi :=\frac{1}{\sqrt{1+2\mu \zeta -\mu^{2}L^{2}}}$*. Then for every* $w\in {\mathbb{R}}^{d}⧵{𝔴}^{*}$ *the following statements hold:*

1. $\left\|ℋ(w)-{𝔴}^{*}\|\leq \Xi \|w-{𝔴}^{*}\right\|$.
2. $\langle w-ℋ(w),{𝔴}^{*}-ℋ(w)\rangle \leq \|w-ℋ(w){\|}^{2}$.
3. $\langle w-{𝔴}^{*},w-ℋ(w)\rangle \geq (1-\Xi )\|w-{𝔴}^{*}{\|}^{2}$.
4. $\left\|w-ℋ(w)\|\geq (1-\Xi )\|w-{𝔴}^{*}\right\|$.

To further highlight the novelty of the proposed framework relative to the most closely related studies, a comparison is presented in Table 1.

**Table 1:** Comparison of the proposed framework with closely related dynamical approaches.

Reference	Problem	FT	PdT	Strongly Pseudomonotone MEP
Ju et al. [36]	EP	✓	×	×
Vuong and Strodiot [38]	EP	×	×	×
Addi K. et al. [39]	VI	✓	×	×
Ju et al. [37]	MVI	×	×	×
Zheng et al. [41]	MVI	×	✓	×
Present paper	Strongly pseudomonotone MEP	✓	✓	✓

Table 1 highlights the main distinctions between the proposed framework and the most closely related studies. Ju et al. [36] established a FT proximal dynamical approach for equilibrium problems, while Vuong and Strodiot [38] investigated dynamical systems for strongly pseudomonotone EPs with asymptotic convergence. Ju et al. and Addi K. et al. [37, 39] and Zheng et al. [41] studied proximal neurodynamic approaches for MVI and VI. In contrast, the present work develops both FT and PdT proximal dynamical systems for strongly pseudomonotone MEPs and establishes the corresponding stability and convergence properties.

## 3. A nominal dynamical system for MEP

To obtain a solution of MEP(Γ,Ω), we formulate the following PDS:


$$\begin{array}{c} \dot{w}=-\sigma (w-ℋ(w)), \end{array}$$ (5)

where σ>0 is a scalar tuning gain. Further, we establish a clear connection between the solutions of the MEP and the equilibrium points of our PDSs. Define the operator $ℋ(w):={\mathrm{prox}}_{\mu \Psi }(w-\mu \Upsilon (w)).$ Then the MEP can be reformulated as the fixed-point problem ${𝔴}^{*}=ℋ({𝔴}^{*})$.

**Theorem 1.** *For any* μ>0*, a point* $w^{*}\in \Omega$ *belongs to* Sol(Γ,Ω) ⟺ ${𝔴}^{*}=ℋ({𝔴}^{*})$

*Proof.* From Lemma 2 (1), $\forall \;{𝔴}^{*}\neq 𝔲\in {\mathbb{R}}^{d}$, we have


$$\begin{array}{cc} {𝔴}^{*} & =ℋ({𝔴}^{*})\\ & ⟺\langle ({𝔴}^{*}-\mu \Upsilon ({𝔴}^{*}))-{𝔴}^{*},𝔲-{𝔴}^{*}\rangle +\mu \Psi ({𝔴}^{*})\leq \mu \Psi (𝔲),\;\forall \;𝔲\in {\mathbb{R}}^{d}. \end{array}$$

This inequality is equivalent to


$$\begin{array}{c} \mu \langle \Upsilon ({𝔴}^{*}),𝔲-{𝔴}^{*}\rangle +\mu \Psi (𝔲)-\mu \Psi ({𝔴}^{*})\geq 0,\;\forall \;𝔲\in {\mathbb{R}}^{d}. \end{array}$$

Dividing both sides by μ>0, we obtain


$$\begin{array}{cc} \langle \Upsilon ({𝔴}^{*}),𝔲-{𝔴}^{*}\rangle +\Psi (𝔲)-\Psi ({𝔴}^{*}) & \geq 0,\;\forall \;𝔲\in {\mathbb{R}}^{d},\\ ⟺\Gamma ({𝔴}^{*},𝔲)\geq 0. \end{array}$$

which completes the proof.

**Remark 5.** *The explicit Euler discretization of PDS (5)*


$$\begin{array}{c} \dot{w}(t)=-\sigma (w(t)-ℋ(w)), \end{array}$$

*By choosing an initial point* $w_{0}\in \Omega$ *with a step size* $h_{n}>0$*, an iterative procedure can be developed as follows:*


$$\begin{array}{c} \frac{w_{n+1}-w_{n}}{h_{n}}=-\sigma \{w_{n}-ℋ(w_{n})\}, \end{array}$$

*or equivalently,*


$$\begin{array}{c} w_{n+1}=(1-\sigma h_{n})w_{n}+\sigma h_{n}ℋ(w_{n}), \end{array}$$ (6)

*This relaxed projection-type method is introduced for solving* MEP(Γ,Ω) *using the relaxation parameter* $\sigma h_{n}$*. The corresponding non-relaxed scheme (i.e.,* $\sigma h_{n}=1$ *for all n) has been studied under strong monotonicity assumptions.*

By standard existence results for strongly pseudomonotone and continuous equilibrium bifunctions defined on a nonempty, closed, and convex set Ω (see, e.g., [34]), the problem MEP(Γ,Ω) admits at least one solution. Moreover, under Assumption 1, the solution is unique. Let ${𝔴}^{*}$ denote this unique solution. By Theorem (1), a point ${𝔴}^{*}$ solves MEP(Γ,Ω) if and only if it is a fixed point of the operator ℋ. Consequently, ${𝔴}^{*}$ is the unique equilibrium point of the proposed dynamical system (5). Therefore, we obtain the following existence and uniqueness result. The existence of a solution follows from [34], while uniqueness follows directly from Remark 3 under Assumption 1.

**Corollary 1.** *Suppose that* Γ *is continuous and satisfies Assumptions 1 and 3 on* Ω*. Then* MEP(Γ,Ω) *admits a solution* ${𝔴}^{*}$. Consequently PDS (5) *possesses a unique equilibrium point* ${𝔴}^{*}$.

We now proceed to prove the global stability of PDS (5). For this purpose, we begin with an estimate that is key to the stability analysis.

**Proposition 2.** *Suppose* $\Omega \text{≠}\emptyset \subseteq {\mathbb{R}}^{d}$*, which is closed and convex. Under Assumptions 1 and 3,* ${𝔴}^{*}$ *be the solution of* MEP(Γ,Ω) *and* μ>0*. Then*


$$\begin{array}{c} \left(1+\mu (2\zeta -\mu L^{2})\right)\|ℋ(w)-{𝔴}^{*}{\|}^{2}\;\leq \;\|w-{𝔴}^{*}{\|}^{2},\;\forall w\in \Omega . \end{array}$$ (7)

*Proof.* By Assumption 3, the bifunction Γ satisfies the Lipschitz-type condition. Therefore, an application of the Cauchy–Schwarz inequality yields, for all w,𝔲,z∈Ω


$$\begin{array}{c} \Gamma (w,𝔲)+\Gamma (𝔲,z)\;\geq \;\Gamma (w,z)-L\;\|w-𝔲\|\;\|𝔲-z\|. \end{array}$$

Using the elementary inequality $\|w-𝔲\|\;\|𝔲-z\|\leq \frac{1}{2\mu L}\|w-𝔲{\|}^{2}+\frac{\mu L}{2}\|𝔲-z{\|}^{2}$, we obtain


$$\begin{array}{c} \Gamma (w,𝔲)+\Gamma (𝔲,z)\;\geq \;\Gamma (w,z)-\frac{1}{2\mu }\|w-𝔲{\|}^{2}-\frac{\mu L^{2}}{2}\|𝔲-z{\|}^{2}. \end{array}$$

Therefore, inequality (7) follows directly from [45, Proposition 4.2].

**Theorem 3.** *Suppose* $\Omega \text{≠}\emptyset \subseteq {\mathbb{R}}^{d}$*, which is closed and convex. Under Assumptions 1, 3, and 4 hold. Then the equilibrium point* ${𝔴}^{*}$ *of* (5) *is globally exponentially stable.*

*Proof.* Suppose the Lyapunov function is


$$\begin{array}{c} 𝔙(w(t))=\frac{1}{2}\;\|w(t)-{𝔴}^{*}{\|}^{2},\;w(t)\in \Omega . \end{array}$$

From the PDS (5), the time derivative of 𝔙 is


$$\begin{array}{c} \dot{𝔙}(t)=\langle w(t)-{𝔴}^{*},\;\dot{w}(t)\rangle =\sigma \;\langle w(t)-{𝔴}^{*},\;ℋ(w)-w(t)\rangle . \end{array}$$

We rewrite the inner product as


$$\begin{array}{c} \dot{𝔙}(t)=\sigma (\langle w(t)-{𝔴}^{*},\;ℋ(w)-{𝔴}^{*}\rangle -\|w(t)-{𝔴}^{*}{\|}^{2}). \end{array}$$

Using the Cauchy–Schwarz inequality together with inequality (7), we obtain


$$\begin{array}{c} \dot{𝔙}(t)\leq \sigma (\|w(t)-{𝔴}^{*}\|\;\|ℋ(w)-{𝔴}^{*}\|-\|w(t)-{𝔴}^{*}{\|}^{2})\leq \sigma \left(\frac{1}{\sqrt{1+\mu (2\zeta -\mu L^{2})}}-1\right)\|w(t)-{𝔴}^{*}{\|}^{2}. \end{array}$$

Define


$$\begin{array}{c} \alpha =\sigma \left(1-\frac{1}{\sqrt{1+\mu (2\zeta -\mu L^{2})}}\right)>0. \end{array}$$

Then, $\dot{𝔙}(t)\leq -\alpha \;\|w(t)-{𝔴}^{*}{\|}^{2}.$ Therefore, integrating the inequality yields, for all *t* > 0,


$$\begin{array}{c} \|w(t)-{𝔴}^{*}\|\leq \|w(0)-{𝔴}^{*}\|\;e^{-\alpha t}. \end{array}$$

Hence, it follows that the equilibrium point ${𝔴}^{*}$ of PDS (5) is globally exponentially stable.

As Γ is strongly pseudomonotone whenever it is strongly monotone, we conclude the following result.

**Corollary 2.** *Suppose* $\Omega \;=\;\;\;\;/\;\emptyset \subseteq {\mathbb{R}}^{d}$*, which is closed and convex. Under Assumptions 1 and 3,* ${𝔴}^{*}$ *be the unique solution of* MEP(Γ,Ω) *and let*


$$\begin{array}{c} \mu \in \left(0,\frac{2\zeta }{L^{2}}\right). \end{array}$$

*Then* ${𝔴}^{*}$ *is globally exponentially stable.*

We proceed to demonstrate the linear convergence of the discrete formulation of PDS (5), specifically the iterative scheme (6).

**Theorem 4.** *We consider all Assumptions of Proposition* (2*) and Assumption 4 hold. Let* $\left\{\sigma_{n}\}\subset [\sigma ,1\right]$ *for some constant* σ>0*, and let* $w_{0}\in \Omega$*. Then the sequence* $\left\{w_{n}\right\}$ *generated by (6)*


$$\begin{array}{c} w_{n+1}=(1-\sigma_{n})w_{n}+\sigma_{n}ℋ(w_{n}). \end{array}$$

*converges linearly to the unique solution* ${𝔴}^{*}$ *of* MEP(Γ,Ω).

*Proof.* Let ${𝔲}_{n}:=ℋ(w_{n})$ and define


$$\begin{array}{c} q=\frac{1}{1+\mu (2\zeta -\mu L^{2})}\in (0,1). \end{array}$$

By inequality (7), we have for all n≥0,


$$\begin{array}{c} \|{𝔲}_{n}-{𝔴}^{*}{\|}^{2}\leq q\;\|w_{n}-{𝔴}^{*}{\|}^{2}. \end{array}$$ (8)

Now compute


$$\begin{array}{c} \|w_{n+1}-{𝔴}^{*}{\|}^{2}=\|(1-\sigma_{n})(w_{n}-{𝔴}^{*})+\sigma_{n}({𝔲}_{n}-{𝔴}^{*}){\|}^{2}. \end{array}$$

Expanding and using (8), we obtain


$$\begin{array}{cc} \|w_{n+1}-{𝔴}^{*}{\|}^{2} & =(1-\sigma_{n})\|w_{n}-{𝔴}^{*}{\|}^{2}+\sigma_{n}\|{𝔲}_{n}-{𝔴}^{*}{\|}^{2}-\sigma_{n}(1-\sigma_{n})\|w_{n}-{𝔲}_{n}{\|}^{2}\\ & \leq (1-\sigma_{n})\|w_{n}-{𝔴}^{*}{\|}^{2}+\sigma_{n}q\|w_{n}-{𝔴}^{*}{\|}^{2}\\ & =\left(1-\sigma_{n}(1-q)\right)\|w_{n}-{𝔴}^{*}{\|}^{2}\\ & \leq \left(1-\sigma (1-q)\right)\|w_{n}-{𝔴}^{*}{\|}^{2}. \end{array}$$

Since 0<1−σ(1−q)<1, the sequence $\left\{w_{n}\right\}$ converges linearly to ${𝔴}^{*}$.

## 4. FT stability analysis

In this section, we study the FT stability of the MEP (2). A first-order PDS is constructed whose equilibrium point corresponds to the solution of (2). Under suitable parameter conditions, the proposed dynamical system is shown to be FT stable.

Before presenting the main results, we recall a well-known characterization of FT stability due to Bhat and Bernstein [27], which will be used to establish the convergence properties of the presented system.

**Lemma 5.** [27] *Consider the dynamical system* (1) *and let* ${𝔴}^{*}\in {\mathbb{R}}^{d}$ *denote one of its equilibrium points. Suppose that* ∃ *a neighborhood* 𝒦 *of* ${𝔴}^{*}$ *and a continuously differentiable Lyapunov function* 𝔙:𝒦→ℝ *s. t.*


$$\begin{array}{c} \dot{𝔙}(w)\leq -\Theta (𝔙(w){)}^{q},\;w\in 𝔘⧵\{{𝔴}^{*}\}, \end{array}$$ (9)

*for some open neighborhood* 𝔘⊆𝒦 *of* ${𝔴}^{*}$, where Θ>0 *and* q∈(0,1)*. Under this condition,* ${𝔴}^{*}$ *is a FT stable equilibrium of (1). Furthermore, the convergence time is bounded by*


$$\begin{array}{c} 𝒯=𝒯(w(0))\leq \frac{𝔙(w(0){)}^{1-q}}{\Theta (1-q)}<\infty \end{array}$$ (10)

*for all* w(0)∈𝔘*. Furthermore, the equilibrium point* ${𝔴}^{*}$ *of (1) is globally FT stable whenever* $𝒦={\mathbb{R}}^{d}$.

To study the FT stability of the MEP (2), we construct another first-order PDS associated with (2), defined as


$$\begin{array}{c} \dot{w}=-\sigma \frac{w-ℋ(w)}{\|w-ℋ(w){\|}^{\frac{\rho -2}{\rho -1}}}, \end{array}$$ (11)

where ρ>2 is a parameter and σ>0.

**Remark 6.** *If* w−ℋ(w)=0, *in PDS* (11), then (11) is s*till well defined owing to* $0<\frac{\rho -2}{\rho -1}<1$*. Indeed, if* w−ℋ(w)=0*, from the right-hand side o*f (11)*, we have*


$$\begin{array}{c} \left\|\frac{w-ℋ(w)}{\|w-ℋ(w){\|}^{\frac{\rho -2}{\rho -1}}}\right\|=\|w-ℋ(w){\|}^{\frac{1}{\rho -1}}=0. \end{array}$$

The following result shows that the equilibrium points of (11) coincide with (5).

**Proposition 5.** *If* ${𝔴}^{*}\in {\mathbb{R}}^{d}$ *be an equilibrium point of (11)* ⟺ *it is also an equilibrium point of (5).*

*Proof.* A necessary and sufficient condition for ${𝔴}^{*}\in {\mathbb{R}}^{d}$ to be an equilibrium point of PDS (11) is that


$$\begin{array}{cc} \frac{{𝔴}^{*}-ℋ({𝔴}^{*})}{\|{𝔴}^{*}-ℋ({𝔴}^{*}){\|}^{\frac{\rho -2}{\rho -1}}}=0 & ⟺\frac{\left\|{𝔴}^{*}-ℋ({𝔴}^{*})\right\|}{\|{𝔴}^{*}-ℋ({𝔴}^{*}){\|}^{\frac{\rho -2}{\rho -1}}}=0\\ & ⟺({𝔴}^{*}-ℋ({𝔴}^{*}){)}^{1-\frac{\rho -2}{\rho -1}}=0. \end{array}$$ (12)

From $1-\frac{\rho -2}{\rho -1}=\frac{1}{\rho -1}>0,\;\forall \rho >2,$ we conclude that equation (12) hold ⟺


$$\begin{array}{c} {𝔴}^{*}-ℋ({𝔴}^{*})=0, \end{array}$$

or


$$\begin{array}{c} {𝔴}^{*}=ℋ({𝔴}^{*}). \end{array}$$

Hence, ${𝔴}^{*}$ is an equilibrium point of (5).

Therefore, the vector field associated with (11) is continuous at the equilibrium point and the finite-time dynamical system is well posed in a neighborhood of $w^{*}$. The result below is an immediate consequence of Corollary 1 together with Proposition 5.

**Corollary 3.** *Suppose* ${𝔴}^{*}$ *be a solution of MEP (2)* ⟺ *it is an equilibrium point of PDS (11).*

*Proof.* It is a direct consequence of Corollary 1 together with Proposition 5.

The following result establishes the FT stability of the PDS (11).

**Theorem 6.** *Suppose Assumptions 1, 3, and 4 hold. Then,* ${𝔴}^{*}\in {\mathbb{R}}^{d}$ *be the soluti*on (2) *is a globally FT stable equilibrium point of PDS (*11) *with a settling time*


$$\begin{array}{c} 𝒯(w(0))\leq \frac{(w(0)-{𝔴}^{*}{)}^{2(1-q)}}{2^{1-q}\Theta (1-q)}, \end{array}$$ (13)

*for some constants* Θ>0 *and* q∈(0.5,1).

*Proof.* We take Lyapunov function $𝔙:{\mathbb{R}}^{d}\to \mathbb{R}$ as:


$$\begin{array}{c} 𝔙(w):=\frac{1}{2}\|w-{𝔴}^{*}{\|}^{2}. \end{array}$$ (14)

Differentiating (14), we obtain


$$\begin{array}{c} \dot{𝔙}(w)=\langle w-{𝔴}^{*},\dot{w}\rangle \end{array}$$

Thus, from any initial condition $w(0)\in {\mathbb{R}}^{d}⧵\{{𝔴}^{*}\}$, where ${𝔴}^{*}\in Zer(M)$ is unique with $M(w)=w-ℋ(w),\;w\in {\mathbb{R}}^{d},$ we obtain


$$\begin{array}{cc} \dot{𝔙} & =-\left\langle w-{𝔴}^{*},\sigma \frac{w-ℋ(w)}{\|w-ℋ(w){\|}^{\frac{\rho -2}{\rho -1}}}\right\rangle \\ & =-\sigma \frac{\langle w-{𝔴}^{*},\;w-ℋ(w)\rangle }{\|w-ℋ(w){\|}^{\frac{\rho -2}{\rho -1}}}. \end{array}$$

By Part (iii) of Lemma 4 it follows that


$$\begin{array}{c} \dot{𝔙}\leq -\sigma (1-\Xi )\frac{\|w-{𝔴}^{*}{\|}^{2}}{\|w-ℋ(w){\|}^{\frac{\rho -2}{\rho -1}}} \end{array}$$

By Part (iv) of Lemma 4, we obtain


$$\begin{array}{cc} \dot{𝔙} & \leq -\sigma (1-\Xi )(1-\Xi {)}^{\frac{\rho -2}{\rho -1}}\frac{\|w-{𝔴}^{*}{\|}^{2}}{\|w-{𝔴}^{*}{\|}^{\frac{\rho -2}{\rho -1}}}\\ & =-\sigma (1-\Xi {)}^{\frac{1}{\rho -1}}\|w-{𝔴}^{*}{\|}^{\frac{\rho }{\rho -1}}. \end{array}$$ (15)

Setting $N:=\sigma (1-\Xi {)}^{\frac{1}{\rho -1}}$, where $\Xi :=\frac{1}{\sqrt{1+2\mu \zeta -\mu^{2}L^{2}}}$. Then condition $\mu L^{2}<2\zeta$ implies that *N* > 0, equation (15) and (14) can be written as


$$\begin{array}{cc} \dot{𝔙} & \leq -N\|w-{𝔴}^{*}{\|}^{\frac{\rho }{\rho -1}}\\ & \leq -N\;2^{\frac{\rho }{2(\rho -1)}}{\left(\frac{1}{2}\|w-{𝔴}^{*}{\|}^{2}\right)}^{\frac{\rho }{2}\frac{1}{\rho -1}}\\ & =-N\;2^{\frac{\rho }{2(\rho -1)}}{𝔙}^{\frac{\rho }{2}\frac{1}{\rho -1}}. \end{array}$$

Applying Lemma 5 and observing that


$$\begin{array}{c} 0.5<q:=\frac{\rho }{2}\frac{1}{\rho -1}<1>2,\text{ with }\Theta :=N\cdot 2^{q}>0, \end{array}$$

the conclusion follows. The proof is complete.

**Remark 7.** *The settling-time estimate in [13] depends explicitly on the initial condition w(0). Therefore, Theorem 6 establishes finite-time stability rather than fixed-time stability.*

## 5. PdT stability analysis

In this section, we introduce a novel PDS designed to solve MEP(Γ,Ω) (2) in a predefined time. Inspired by research on PdT convergence for multi-agent distributed optimization problems [31], we introduce the following PdT converging PDS:


$$\begin{array}{c} \dot{w}=-\frac{1}{2s\pi {𝒯}_{c}}\exp\;\left(\|w-ℋ(w){\|}^{2s}\right)\frac{\left(w-ℋ(w)\right)}{\|w-ℋ(w){\|}^{2s}}. \end{array}$$ (16)

where ${𝒯}_{c}>0$, $0<s<\frac{1}{2}$, and π=(1−Ξ)∈(0,1).

**Lemma 6.** *The point* ${𝔴}^{*}$ *is an equilibrium point for the PDS (16)* ⟺*it is an equilibrium point of system (5).*

*Proof.* If ${𝔴}^{*}\in {\mathbb{R}}^{d}$ is an equilibrium point PDS (16), i.e.,


$$\begin{array}{cc} \dot{w} & =0\\ \Rightarrow & -\frac{1}{2s\pi {𝒯}_{c}}\exp\left(\|{𝔴}^{*}-ℋ({𝔴}^{*}){\|}^{2s}\right)\frac{{𝔴}^{*}-ℋ({𝔴}^{*})}{\|{𝔴}^{*}-ℋ({𝔴}^{*}){\|}^{2s}}=0\\ \Rightarrow {𝔴}^{*} & =ℋ({𝔴}^{*}). \end{array}$$

This indicates that ${𝔴}^{*}$ constitutes an equilibrium point for system (5). The reverse assertion follows analogously, thereby concluding the proof. □

The result below is an immediate consequence of Corollary (1) together with Lemma (6).

**Corollary 4.** *Let* ${𝔴}^{*}$ *be a solution of* MEP(Γ,Ω) *(2)* ⟺ *it is an equilibrium point of the PDS (16).*

Based on Theorem (1), it is established that the equilibrium point of system (5) corresponds precisely to the solution ${𝔴}^{*}$ of the MEP(Γ,Ω) (2). Moreover, as per Lemma (6), it is also established that the equilibrium point ${𝔴}^{*}$ of PDS (16) coincides with the solution ${𝔴}^{*}$ of MEP(Γ,Ω) (2).

**Theorem 7.** *Let* ${𝔴}^{*}$ *be the solution to the* MEP(Γ,Ω) *(2). Under Assumptions 1, 3 and 4 the solution trajectories of PDS (16) converge to the equilibrium point* ${𝔴}^{*}$ *in predefined time* ${𝒯}_{c}$*.*

*Proof.* We consider the Lyapunov function


$$\begin{array}{c} 𝔙(w)=\|w-{𝔴}^{*}{\|}^{2}. \end{array}$$ (17)

Differentiating (17) along the trajectories of (16), we obtain


$$\begin{array}{c} \begin{array}{cc} \dot{V}(w) & =2\langle w-w^{*},\dot{w}\rangle \\ & =\left(-\frac{1}{s\pi T_{c}}\right)\exp\;\left(\|w-H(w){\|}^{2s}\right)\frac{\langle w-w^{*},\;w-H(w)\rangle }{\|w-H(w){\|}^{2s}}. \end{array} \end{array}$$

Using the Cauchy-Schwarz inequality together with Lemma (6) and Corollary (4), which states that $w^{*}$ is the equilibrium point of PDS (16), we obtain


$$\begin{array}{c} \begin{array}{cc} \dot{V}(w) & \leq \left(-\frac{1}{s\pi T_{c}}\right)\exp\;\left(\|w-H(w){\|}^{2s}\right)\|w-w^{*}\|\frac{\left\|w-H(w)\right\|}{\|w-H(w){\|}^{2s}}\\ \dot{V}(w) & \leq -\frac{1}{s\pi T_{c}}\exp\;\left(\|w-H(w){\|}^{2s}\right)\|w-w^{*}\|\|w-H(w){\|}^{1-2s}. \end{array} \end{array}$$

Now apply Lemma (4) in the above inequality, we obtain


$$\begin{array}{c} \begin{array}{c} \dot{V}(w)\leq -\frac{(1-\Xi {)}^{1-2s}}{s\pi T_{c}}\exp\;\left((1-\Xi {)}^{2s}\|w-w^{*}{\|}^{2s}\right)\|w-w^{*}{\|}^{1-2s}. \end{array} \end{array}$$

From equation (17) and π=(1−Ξ)∈(0,1), we obtain


$$\begin{array}{c} \begin{array}{cc} \dot{V}(w) & \leq -\frac{1}{s(1-\Xi {)}^{2s}T_{c}}\exp\;\left((1-\Xi {)}^{2s}V(w{)}^{s}\right)V(w{)}^{\frac{1-2s}{2}}. \end{array} \end{array}$$

where $0<p=s<\frac{1}{2},\lambda =(1-\Xi {)}^{2s}>0$, $q=\frac{1-2s}{2}\in (0,1)$. Hence, the above differential inequality (5) satisfies the conditions of Lemma (3). Therefore, the equilibrium point $w^{*}$ of PDS (16) is predefined-time stable, and every trajectory converges to $w^{*}$ within the prescribed time $T_{c}$, where ${𝒯}_{c}$ serves as an a priori upper bound of the settling time.□

## 6. Numerical Examples: MEP

In this section, we illustrate the effectiveness of the proposed PDSs (5) and (16) for solving the MEP. Simulations are performed in MATLAB R2024a with an Intel(R) Core(TM) i5 processor and 16.00 GB RAM.

**Example 1.** *Let* w,𝔲∈Ω*. We consider a bifunction as*


$$\begin{array}{c} \Gamma (w,𝔲)=\langle (P+Q)w+r,\;𝔲-w\rangle +\Psi (𝔲)-\Psi (w), \end{array}$$

*where* $\Psi :{\mathbb{R}}^{5}\to \mathbb{R}$ *is given by* $\Psi (w)=\frac{1}{2}w^{⊤}Mw.$ *The vectors and matrices are defined as follows:*


$$\begin{array}{c} r=(\begin{array}{c} 2\\ -1\\ -1.5\\ 3\\ -2 \end{array}),\;P=(\begin{array}{ccccc} 4.2 & 2 & 0 & 0 & 0\\ 3 & 4.3 & 0 & 0 & 0\\ 0 & 0 & 4.4 & 3 & 0\\ 0 & 0 & 3 & 4.5 & 0\\ 0 & 0 & 0 & 0 & 4 \end{array}),\;Q=(\begin{array}{ccccc} 2.6 & 2 & 0 & 0 & 0\\ 2 & 2.6 & 0 & 0 & 0\\ 0 & 0 & 2.5 & 2 & 0\\ 0 & 0 & 2 & 2.5 & 0\\ 0 & 0 & 0 & 0 & 3 \end{array}). \end{array}$$

The regularization matrix is chosen as *M = 3 I*_*5*_*, where I*_*5*_ *denotes the* 5×5 *identity matrix. The matrix P + Q is symmetric and positive definite. Let* $\zeta_{\Upsilon }=\lambda_{\min}(P+Q),\;\zeta_{\Psi }=\lambda_{\min}(M)=3,$ *and* $\zeta =\zeta_{\Upsilon }+\zeta_{\Psi }.$ $L_{\Upsilon }=\|P+Q{\|}_{2},$ *and for* ∇Ψ(w)=Mw, $L_{\Psi }=\|M{\|}_{2}=3.$ *Hence, the total Lipschitz constant is* $L=L_{\Upsilon }+L_{\Psi }.$ *where the feasible set is* $\Omega =\left\{w\in {\mathbb{R}}^{5}\;|\;\sum_{i=1}^{5}w_{i}\geq 0,\;-15\leq w_{i}\leq 15\right\}.$ *The continuous-time PDS associated with the MEP is simulated with the parameters chosen as* $\mu =\frac{1.9\zeta }{L^{2}},\;\sigma =0.7.$ *The initial condition is* $w(0)=(1.8,\;1,\;3.9,\;-1,\;-4{)}^{⊤}.$

**Fig 1:**
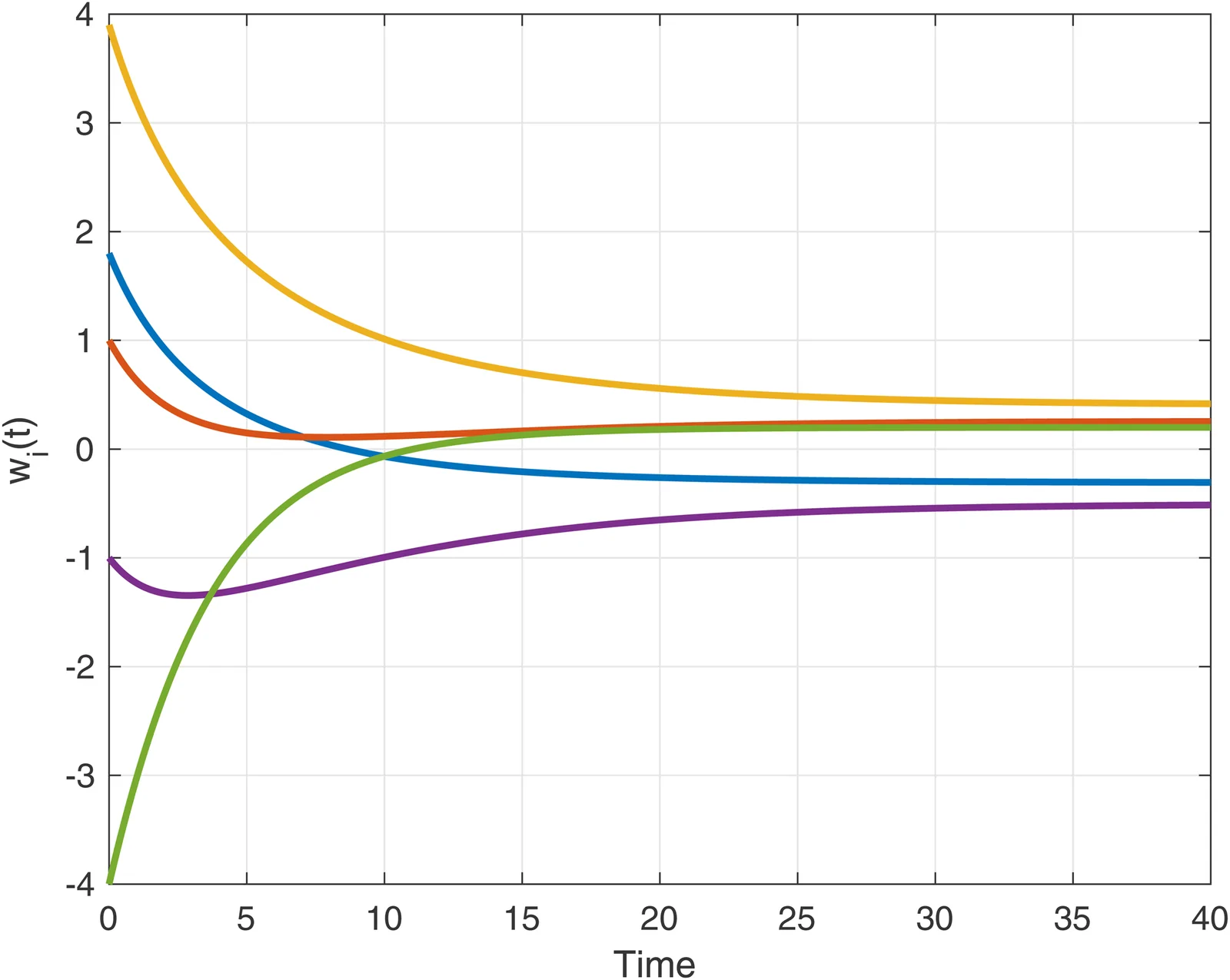
Trajectory of the system (5) for σ=0.7 with initial condition $w_{0}=(1.8,\;1,\;3.9,\;-1,\;-4{)}^{⊤}$.

*It is observed that in* Fig 1 *the state w(t) converges exponentially toward the unique equilibrium point* ${𝔴}^{*}=(-0.309010,0.257076,0.405409,-0.502706,0.2000)$.

**Fig 2:**
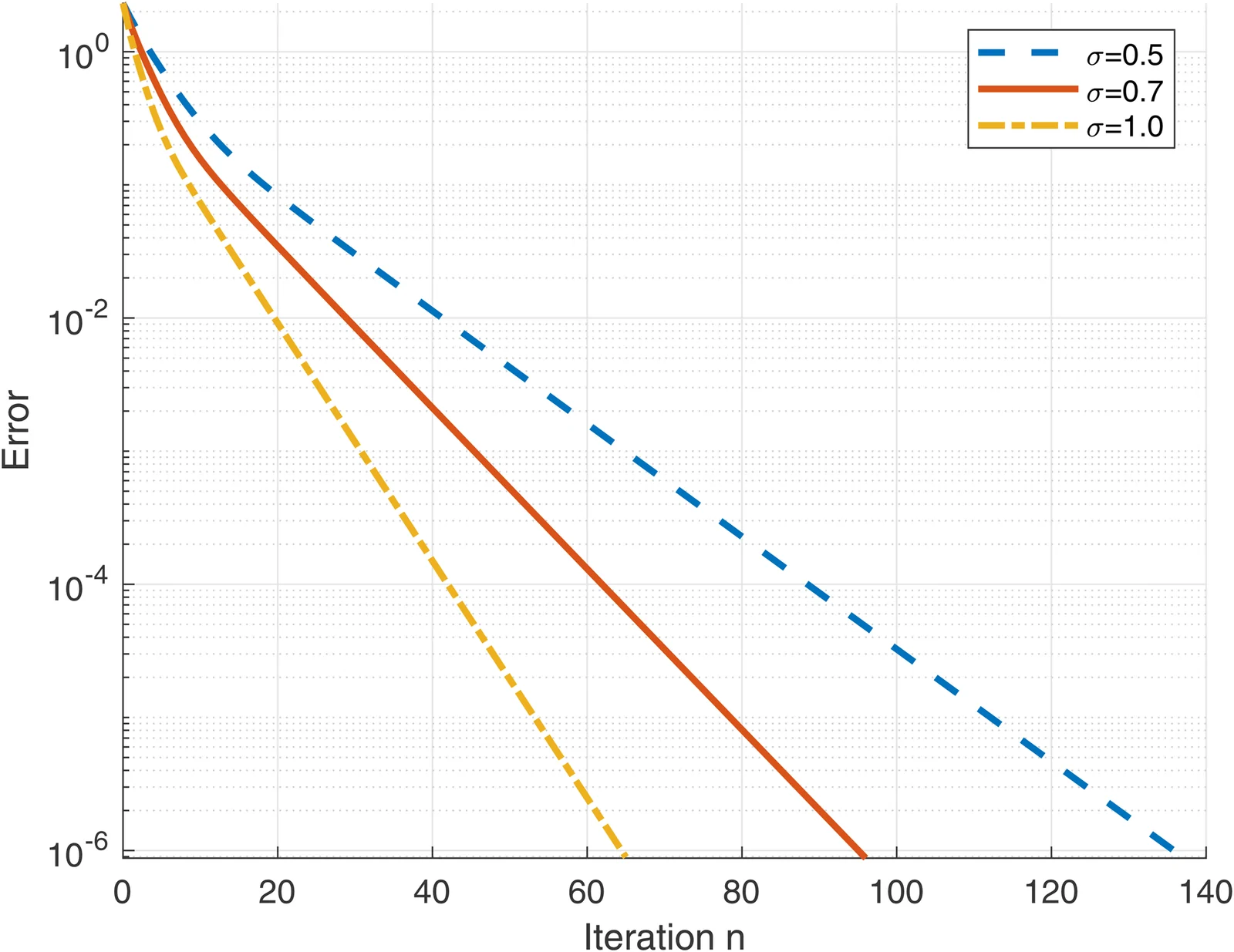
Convergence profiles of the proposed algorithm for $\sigma_{n}=0.5,;0.7,;1.0$ with initial condition ${𝔴}_{0}=(1.8,;1,;3.9,;-1,;-4{)}^{⊤}$.

The convergence behavior of the proposed algorithm for different values of the relaxation parameter σ is reported in Table 2. The simulation parameters were selected to satisfy the theoretical stability conditions. In particular, μ was chosen according to Assumption 4, i.e., $\mu L^{2}<2\zeta$. As shown in Fig 2 and Table 2, increasing σ reduces the number of iterations required to reach the equilibrium solution ${𝔴}^{*}$. All tested values converge to the same equilibrium point, indicating that σ mainly affects the convergence rate while preserving the solution of the mixed equilibrium problem.

**Table 2:** Convergence behavior for different values of σ.

$\sigma_{n}=\sigma$	Number of iterations	Final Equilibrium Point ${𝔴}^{*}$
0.5	136	$\left(\begin{array}{ccccc} -0.309010 & 0.257076 & 0.405409 & -0.502706 & 0.2000 \end{array}\right)$
0.7	96	$\left(\begin{array}{ccccc} -0.309010 & 0.257076 & 0.405409 & -0.502706 & 0.2000 \end{array}\right)$
1.0	65	$\left(\begin{array}{ccccc} -0.309010 & 0.257076 & 0.405408 & -0.502706 & 0.2000 \end{array}\right)$

**Example 2.** *Here, we consider a bifunction* Γ:Ω×Ω→ℝ *is defined by*


$$\begin{array}{c} \Gamma (w,𝔲)=\langle Aw+Bw+c,\;𝔲-w\rangle +\Psi (𝔲)-\Psi (w),\;\forall w,𝔲\in {\mathbb{R}}^{5}, \end{array}$$ (18)

*where matrices* $A,B\in {\mathbb{R}}^{5\times 5}$ *and the vector* $c\in {\mathbb{R}}^{5}$ *are defined as*


$$\begin{array}{c} A=(\begin{array}{ccccc} 3.2 & 0.8 & 0 & 0 & 0\\ 0.6 & 2.9 & 0.5 & 0 & 0\\ 0 & 0.4 & 2.7 & 0.6 & 0\\ 0 & 0 & 0.5 & 2.6 & 0.4\\ 0 & 0 & 0 & 0.3 & 2.4 \end{array}),\;B=(\begin{array}{ccccc} 2.8 & 0.6 & 0 & 0 & 0\\ 0.5 & 2.7 & 0.4 & 0 & 0\\ 0 & 0.3 & 2.6 & 0.4 & 0\\ 0 & 0 & 0.3 & 2.5 & 0.3\\ 0 & 0 & 0 & 0.2 & 2.3 \end{array}), \end{array}$$

*and* $c=(1.8,\;-1.2,\;0.9,\;-0.7,\;1.1{)}^{⊤}.$ *The feasible set* Ω *is defined as*


$$\begin{array}{c} \Omega =\left\{w\in {\mathbb{R}}^{5}\;|\;\sum_{i=1}^{5}w_{i}\geq 1,\;-15\leq w_{i}\leq 15,\;i=1,...,5\right\}. \end{array}$$

*The function* $\Psi :{\mathbb{R}}^{5}\to \mathbb{R}\cup \{+\infty \}$ *is chosen as the indicator function of* Ω*, i.e.,*


$$\begin{array}{c} \Psi (w)=\left\{ \begin{array}{cc} 0, & w\in \Omega ,\\ +\infty , & \text{otherwise}. \end{array}\right. \end{array}$$

**Fig 3:**
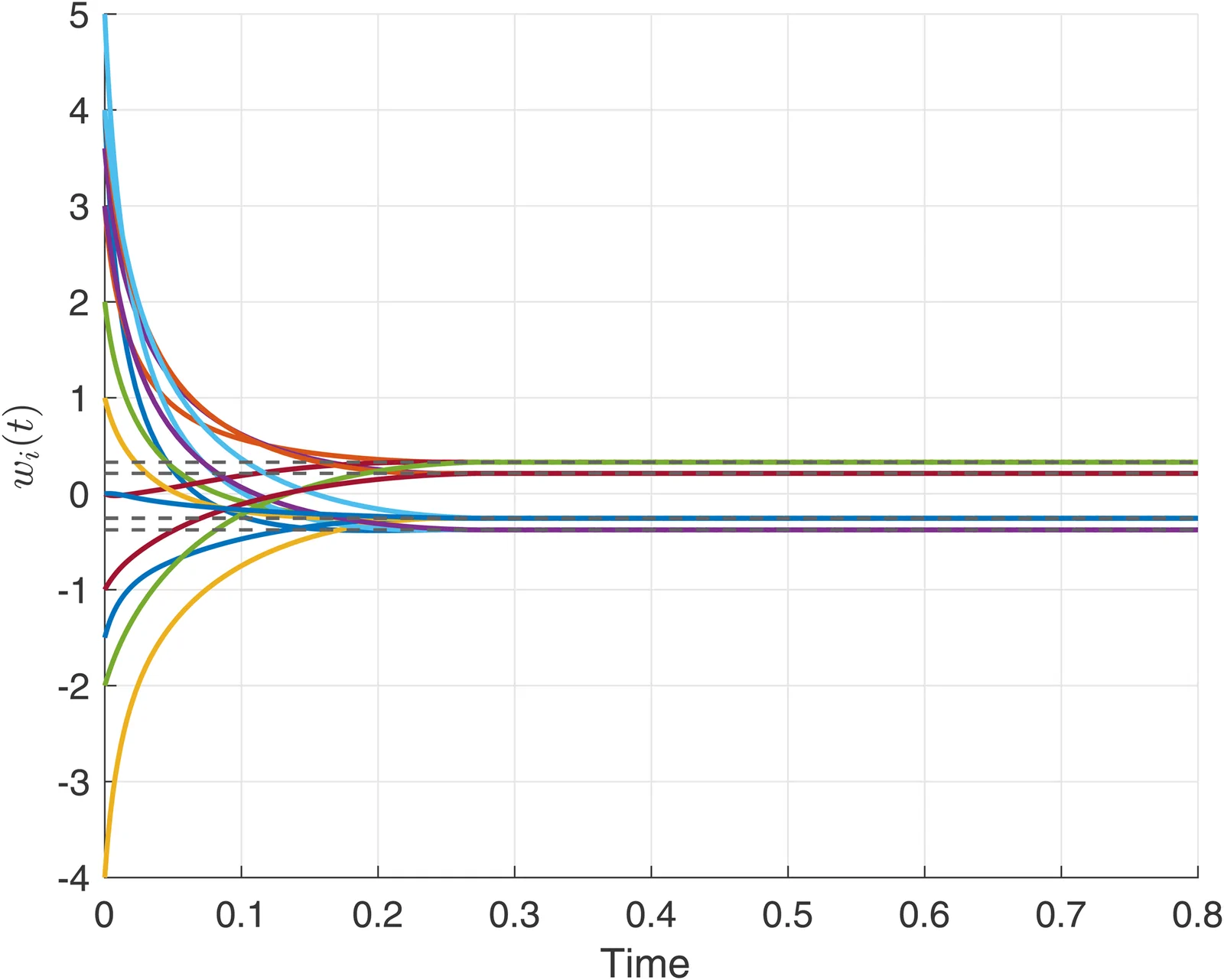
State trajectories of the PDS (16) converging to the equilibrium point ${𝔴}^{*}$ within the PdT ${𝒯}_{c}=0.8$.

**Fig 4:**
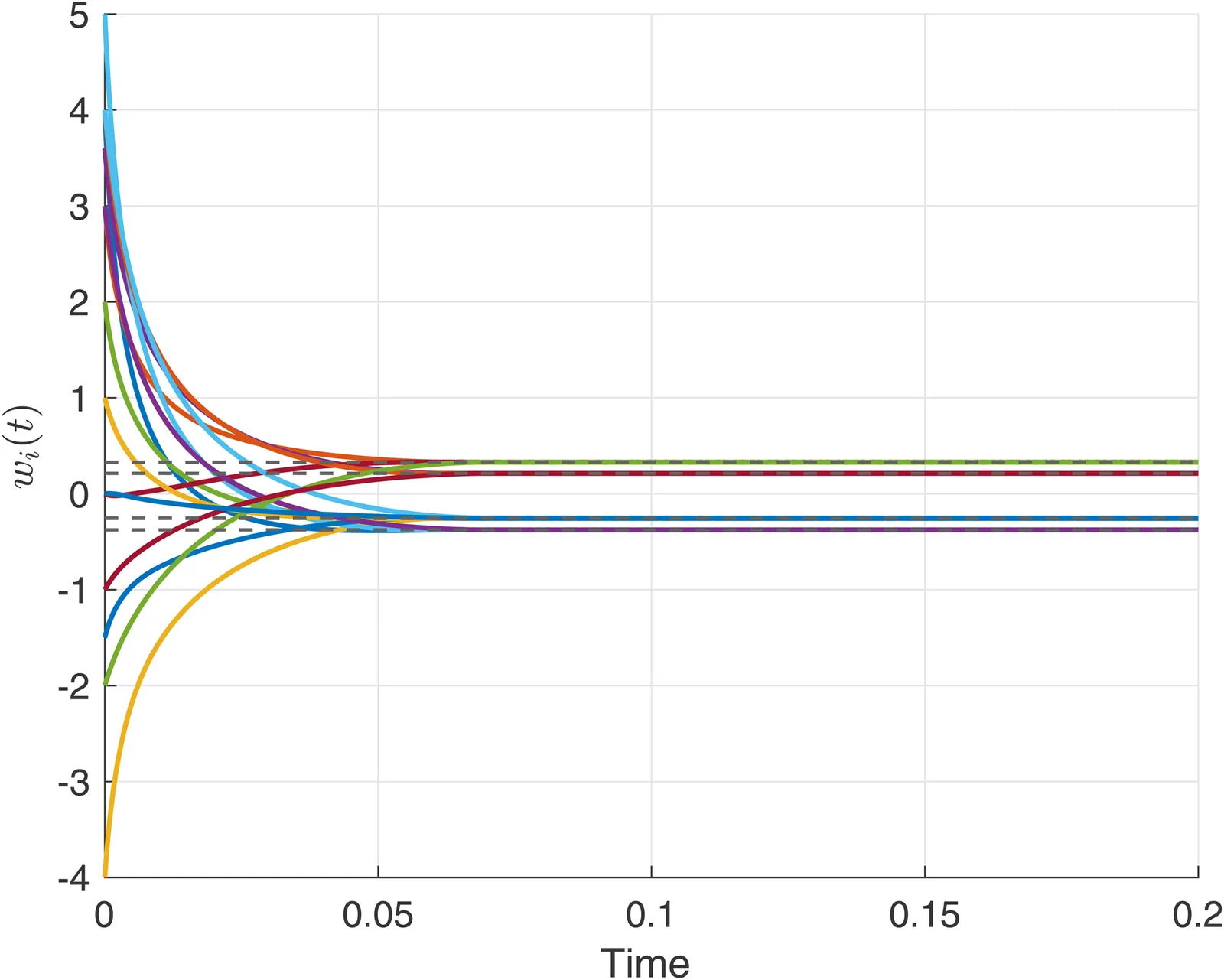
State trajectories of the PDS (16) converging to the equilibrium point ${𝔴}^{*}$ within the PdT ${𝒯}_{c}=0.2$.

*The parameters are selected as* $\mu =0.2,\;s=0.3,\;\pi =0.3,\;{𝒯}_{c}=0.8$ *for* Fig 3*. and* ${𝒯}_{c}=0.2$ *for* Fig 4*. Numerical computation yields the equilibrium point* ${𝔴}^{*}=(-0.3768,\;0.3290,\;-0.2533,\;0.2122,\;-0.2566{)}^{⊤}$ *which also constitutes a solution of the MEP associated with the proposed PdT-PDS.*

**Fig 5:**
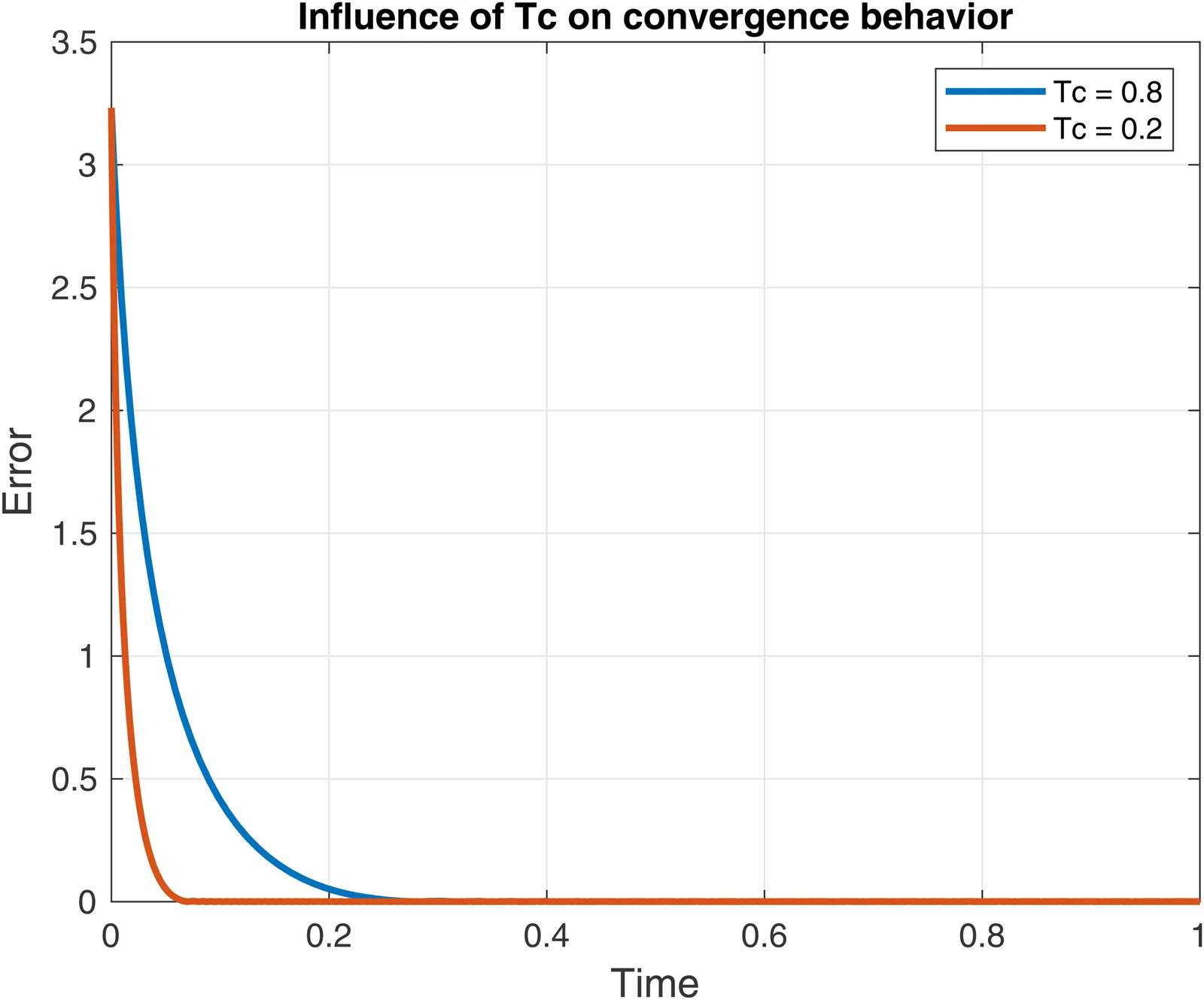
Influence of the predefined-time parameter ${𝒯}_{c}$ on the convergence behavior of the proposed PdT-PDS. Smaller values of $\begin{array}{c} {𝒯}_{c} \end{array}$ lead to faster convergence toward the equilibrium solution.

*Therefore, it constitutes a solution of the MEP associated with the PDS* (16)*. As illustrated in* Fig 3 and Fig 4, *all trajectories of the proposed PdT-PDS converge to the same equilibrium point* ${𝔴}^{*}$. The state trajectories are presented for two different values of the predefined-time parameter, namely ${𝒯}_{c}=0.8$ *and* ${𝒯}_{c}=0.2$*, to illustrate the influence of* ${𝒯}_{c}$ *on the convergence behavior of the proposed system.* Fig 5 *further demonstrates that smaller values of* ${𝒯}_{c}$ *lead to faster convergence toward the equilibrium solution. The convergence behavior corresponding to different values of* ${𝒯}_{c}$ *is also summarized in* Table 3*, which is consistent with the theoretical analysis. These numerical experiments further indicate that the proposed PdT framework preserves stability and convergence behavior under moderate parameter variations, thereby demonstrating the robustness of the presented scheme.*

**Table 3:** Sensitivity of convergence behavior with respect to ${𝒯}_{c}$.

${𝒯}_{c}$	Observed convergence speed	Stability	Final Equilibrium Point ${𝔴}^{*}$
0.8	Moderate	Stable	$\left(\begin{array}{ccccc} -0.3768 & 0.3290 & -0.2533 & 0.2122 & -0.2566 \end{array}\right)$
0.2	Very fast	Stable	$\left(\begin{array}{ccccc} -0.3768 & 0.3290 & -0.2533 & 0.2122 & -0.2566 \end{array}\right)$

### 6.1. Error convergence response

**Fig 6:**
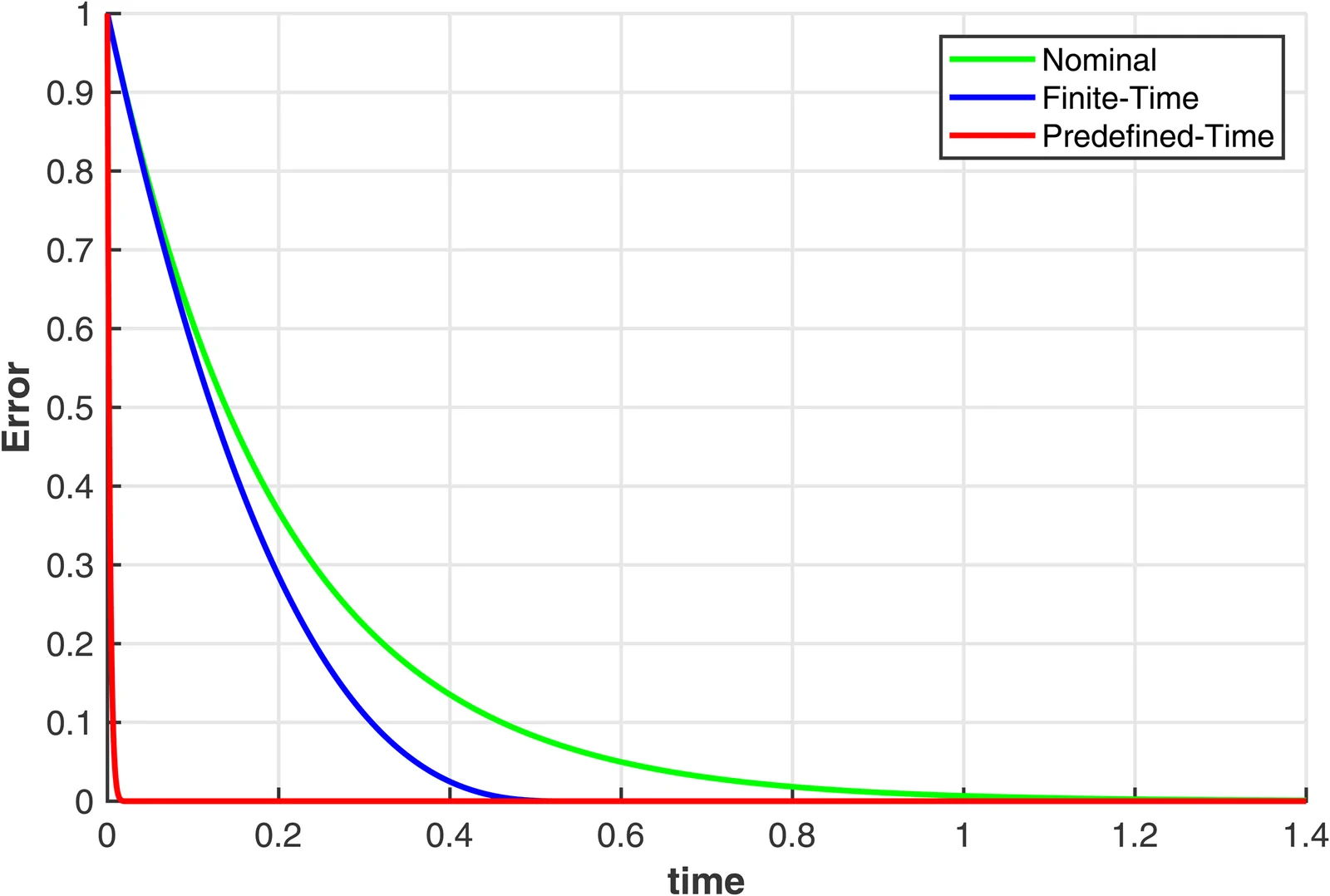
Error convergence comparison between the nominal, FT, and PdT PDS.

In this subsection, we investigate the error convergence behavior of the PdT PDS (16) and compare it with the FT PDS (11) and nominal PDS (5), as illustrated in Fig 6. The convergence times and corresponding speedup factors of the considered methods are summarized in Table 4.

**Table 4:** Convergence Time Comparison of Different Methods.

Method	Convergence Time (s)	Speedup Factor
PDS (PdT) (16)	0.0165	1.0× (baseline)
Finite-Time (FT) (11)	0.4943	29.96× slower
Nominal PDS (5)	1.3830	83.84× slower

Instead of simulating the full MEP (2) involving proximal or projection operators, we analyze the scalar error convergence dynamics derived from the corresponding Lyapunov stability analysis. This simplified representation is sufficient for comparing the convergence speed of different dynamical systems and is widely used in the related literature.

The PdT PDS (16) follows the error dynamics induced by the PdT PDS (16) with parameters ${𝒯}_{c}=0.2$, π=0.2, and *s* = 0.1. For comparison, the FT PDS (11) is implemented with parameters σ=5 and ρ=2.6, satisfying the condition ρ>2 required for FT stability. In addition, a nominal system (5) is included as a baseline method.

The initial error is set to *e*(0)=1, and the simulation time interval is chosen as [0, 1.4]. The convergence threshold is selected as 10^−3^ to evaluate the convergence time of each method. The numerical results demonstrate that the PdT PDS achieves the fastest convergence, followed by the FT method, while the nominal system exhibits the slowest exponential decay. This comparison highlights the advantage of the PdT framework in guaranteeing rapid and predictable convergence.

### 6.2. Application to signal recovery

In this section, we demonstrate the effectiveness of the proposed PdT-PDS (16) for sparse signal recovery within the framework of compressed sensing. The measurement model is given by


$$\begin{array}{c} 𝐲=𝐁w+\varepsilon , \end{array}$$ (19)

where $𝐁\in {\mathbb{R}}^{m\times d}$ is a sensing matrix with Gaussian entries scaled by $1/\sqrt{m}$, $w\in {\mathbb{R}}^{d}$ is the unknown sparse signal, and $\varepsilon \sim 𝒩(0,\sigma^{2}I)$ represents additive Gaussian noise. The sparse recovery problem is formulated as


$$\begin{array}{c} \min_{w\in {\mathbb{R}}^{d}}\frac{1}{2}\|𝐁w-𝐲{\|}_{2}^{2}\;\text{subject to}\;\|w{\|}_{1}\leq K, \end{array}$$ (20)

where *K* > 0 controls the sparsity level. The gradient of the data-fidelity term is defined as $\Upsilon (w)={𝐁}^{T}(𝐁w-𝐲)$, and the projection operator onto the $\ell_{1}$-ball is $ℋ(w)={proj}_{\Omega }(w-\mu \Upsilon (w)),\;\Omega =\{w\in {\mathbb{R}}^{d}:\|w{\|}_{1}\leq K\}$, where μ>0 is a step-size parameter. For numerical implementation of the presented system (16), we employ an explicit Euler discretization:


$$\begin{array}{c} w_{d+1}=w_{d}-h\;\phi (\|r_{d}\|)\;r_{d}, \end{array}$$ (21)

where


$$\begin{array}{c} r_{d}=w_{d}-ℋ(w_{d}),\text{ and }\phi (\|r_{d}\|)=\frac{1}{2s\pi {𝒯}_{c}}\frac{\exp(\|r_{d}{\|}^{2s})}{\|r_{d}{\|}^{2s}+\varepsilon } \end{array}$$

with ε>0 ensuring numerical stability.

**Fig 7:**
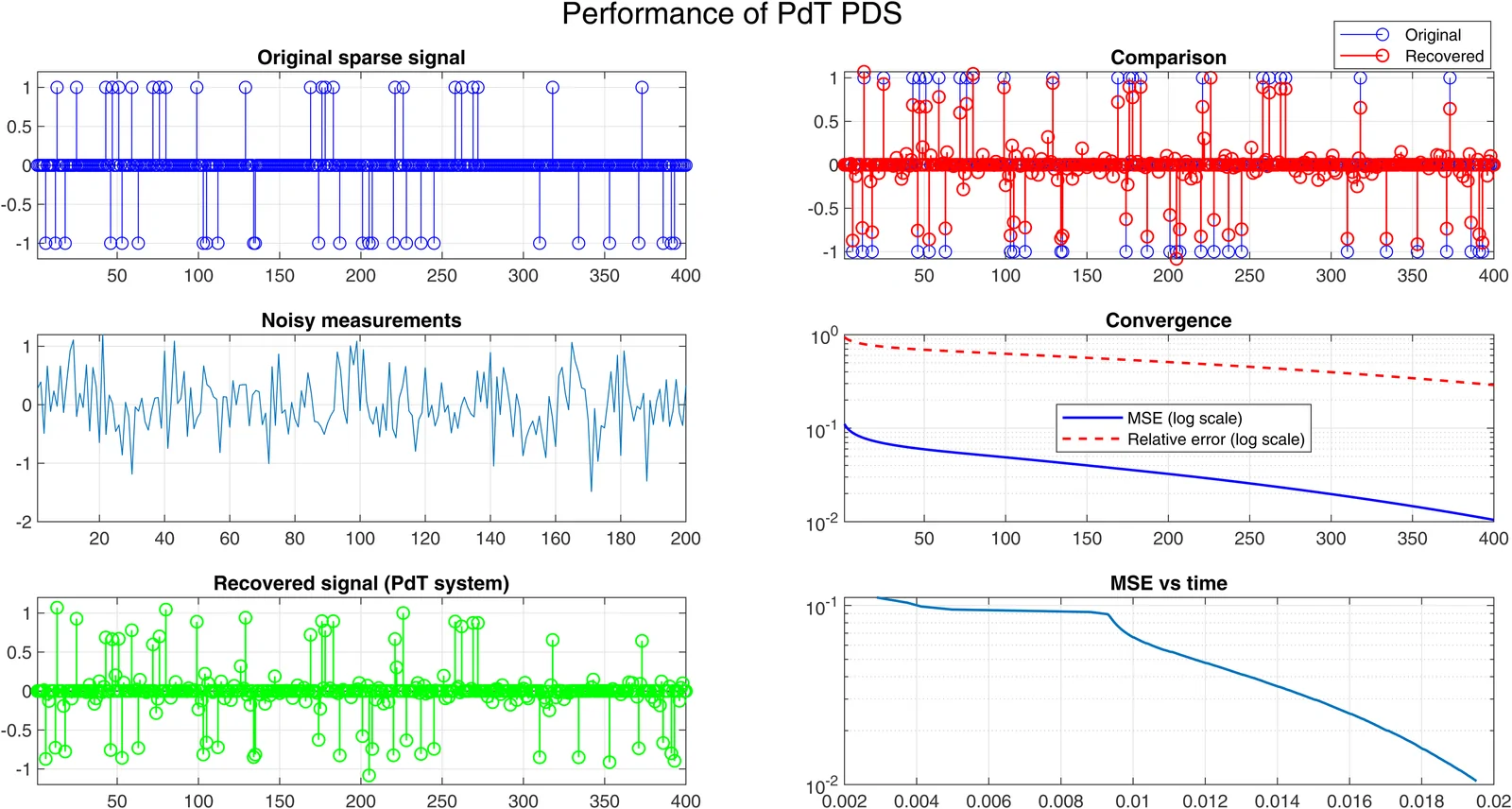
Reconstruction performance of the proposed PdT-PDS method. The left column shows the original sparse signal, noisy measurements, and recovered signal, The right column presents comparison plots, convergence behavior of MSE and relative error (log scale), and MSE versus time.

As shown in Fig 7, the recovered signal closely matches the original sparse signal and accurately identifies both the support and amplitudes of the nonzero entries. These results demonstrate the robustness of the proposed PdT-PDS method in the presence of measurement noise.

#### 6.2.1. Comparison methods.

The iterative scheme (21) is used to compute the solution. To assess the performance of the developed method, we compare it with three benchmark algorithms. In particular, we include the projected gradient (PG) method, which solves the same $\ell_{1}$-ball constrained sparse recovery problem considered in this work. Furthermore, ISTA and FISTA are included as widely used benchmark algorithms for sparse signal recovery based on the corresponding $\ell_{1}$-regularized formulation.

**Projected Gradient (PG) Method:** We consider the projected gradient (PG) method, which solves the same $\ell_{1}$-ball constrained sparse recovery problem. Starting from an initial point *w*_0_, the PG iteration is given by


$$\begin{array}{c} w_{k+1}=P_{\Omega }\left(w_{k}-\frac{1}{L}\nabla \Psi (w_{k})\right), \end{array}$$

where *L* denotes the Lipschitz constant of the gradient ∇Ψ, and $P_{\Omega }$ represents the Euclidean projection onto the $\ell_{1}$-ball $\Omega =\left\{w\in {\mathbb{R}}^{n}:\|w{\|}_{1}\leq K\right\}$.

Unlike ISTA and FISTA, which solve the corresponding $\ell_{1}$-regularized optimization problem, the PG method directly handles the same constrained formulation as the proposed PdT-PDS framework. Therefore, the inclusion of PG provides a more equitable benchmark for evaluating the performance of the proposed method.

**ISTAShrinkage (Iterative -Thresholding Algorithm):** ISTA solves the $\ell_{1}$-regularized problem


$$\begin{array}{c} \min_{w}\frac{1}{2}\|𝐁w-𝐲{\|}_{2}^{2}+\lambda \|w{\|}_{1}, \end{array}$$

where λ>0 controls the sparsity level. The corresponding iterative update is


$$\begin{array}{c} w_{d+1}=soft(w_{d}-\alpha \Upsilon (w_{d}),\lambda \alpha ), \end{array}$$ (22)

where α=1/L with $L=\|𝐁{\|}^{2}$. The soft-thresholding operator is applied component-wise as $\left[soft(x,\tau ){]}_{i}=sign(x_{i})\max(|x_{i}|-\tau ,0\right)$ with $x=w_{d}-\alpha \Upsilon (w_{d})$ and τ=λα.

**FISTA (Fast ISTA):** FISTA improves ISTA by incorporating a Nesterov-type acceleration scheme:


$$\begin{array}{c} w_{d+1}=soft(z_{d}-\alpha \Upsilon (z_{d}),\lambda \alpha ), \end{array}$$ (23)

where $z_{d}=w_{d}+\beta_{d}(w_{d}-w_{d-1})$. We consider *d* = 400, *m* = 200, and sparsity level *K* = 50. The algorithm parameters are $\mu =0.8,\;h=0.02,\;s=0.3,\;\pi =0.9,\;{𝒯}_{c}=0.5$. All simulations were performed using MATLAB R2024a. The sensing matrix $B\in {\mathbb{R}}^{m\times n}$ was generated from a standard Gaussian distribution, and a fixed random seed (rng(1)) was used to ensure reproducibility. The sparse signal contained (K = 50) nonzero components, while Gaussian noise with standard deviation (0.01) was added to the measurements. The performance of the proposed method was evaluated in terms of the mean squared error (MSE) and the relative reconstruction error (RelErr), defined by


$$\begin{array}{c} MSE=\frac{1}{n}\|w-w_{true}{\|}_{2}^{2},\;RelErr=\frac{\|w-w_{true}{\|}_{2}}{\|w_{true}{\|}_{2}}. \end{array}$$

For comparison purposes, we additionally implemented the projected gradient (PG) method, which solves the same $\ell_{1}$-ball constrained sparse recovery problem considered in this work. Furthermore, ISTA and FISTA were included as widely used benchmark algorithms for sparse signal recovery.

Table 5 presents the reconstruction performance of the proposed PdT-PDS, PG, ISTA, and FISTA methods. The proposed PdT-PDS achieves the lowest MSE and relative reconstruction error, demonstrating its superior performance for sparse signal recovery.

**Table 5:** Performance comparison of the proposed PdT-PDS, PG, ISTA, and FISTA methods for sparse signal recovery.

Method	MSE	Relative Error	Time (s)
PdT-PDS (Proposed)	$1.3547\times 10^{-4}$	$3.2920\times 10^{-2}$	0.0503
PG	$2.8379\times 10^{-3}$	$1.5068\times 10^{-1}$	0.4125
ISTA	$4.9161\times 10^{-3}$	$1.9832\times 10^{-1}$	1.0302
FISTA	$4.8938\times 10^{-3}$	$1.9786\times 10^{-1}$	0.9501

**Fig 8:**
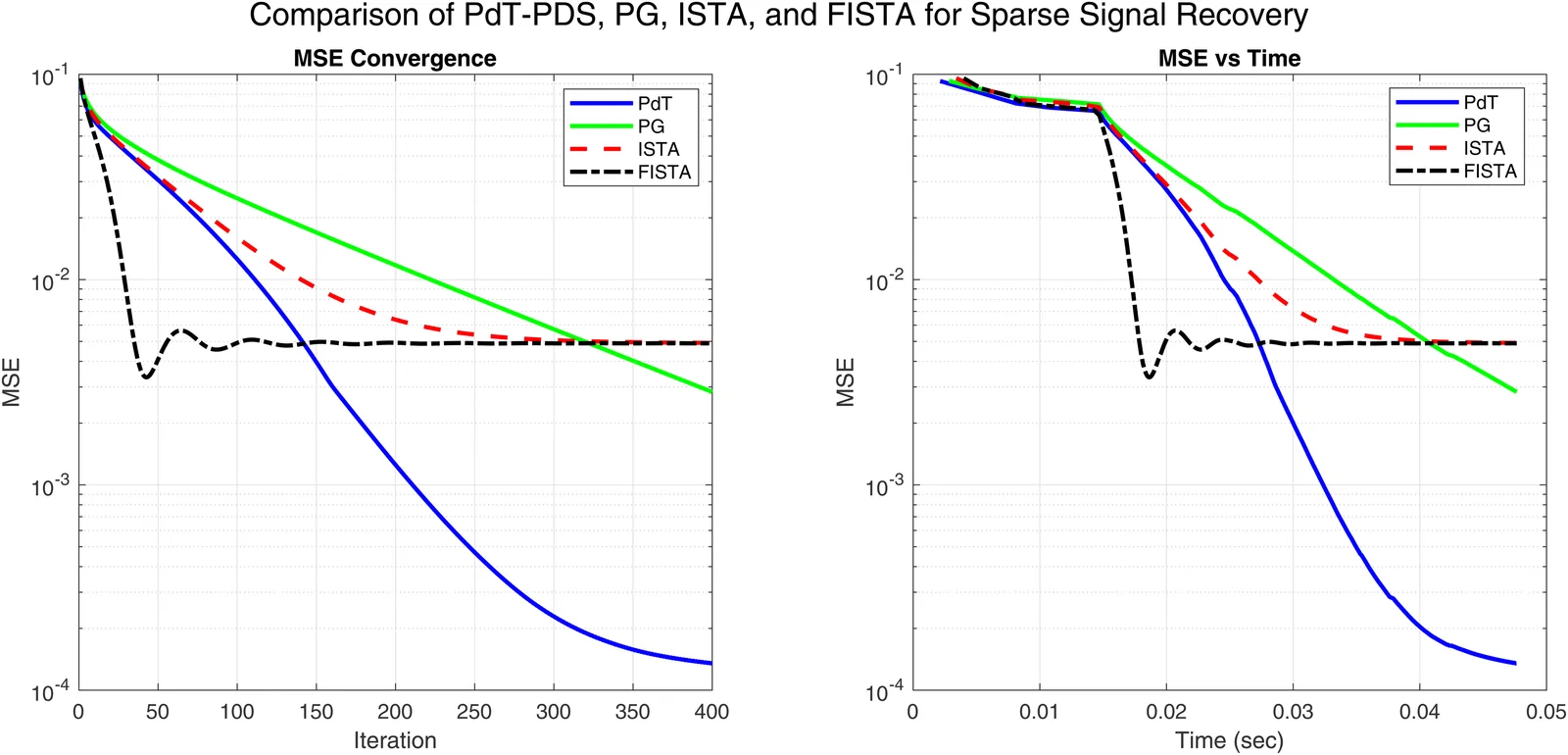
Comparison of convergence behavior for PdT-PDS, PG, ISTA, and FISTA. The proposed PdT-PDS exhibits significantly faster error decay and achieves the lowest reconstruction error.

Fig 8 compares the convergence profiles of the proposed PdT-PDS, PG, ISTA, and FISTA methods. The proposed PdT-PDS converges faster and achieves a lower reconstruction error than the competing methods. These results demonstrate the effectiveness of the proposed method for sparse signal recovery.

## 7. Conclusion

In this work, we investigated proximal dynamical system (PDS) approaches for solving strongly pseudomonotone mixed equilibrium problems (MEPs) in Hilbert spaces. Under suitable assumptions, the presented nominal PDS was shown to admit a unique equilibrium solution that is globally exponentially stable. A discrete-time realization of the system led to a proximal iterative algorithm with linear convergence. Furthermore, two proximal dynamical models with finite-time (FT) and predefined-time (PdT) stability properties were developed. The FT model guarantees convergence to the equilibrium solution within finite time, whereas the PdT model ensures convergence within a user-prescribed time independent of the initial state. These results extend finite-time and predefined-time stability concepts to the class of strongly pseudomonotone MEPs. Numerical experiments verified the theoretical findings and demonstrated that the proposed PdT framework achieves faster and more predictable convergence than the nominal and FT models. In addition, the sparse signal recovery application illustrated the effectiveness and robustness of the proposed approach, where the PdT-PDS outperformed PG, ISTA, and FISTA in terms of reconstruction accuracy.

Future work will focus on extending the presented framework to pseudomonotone and nonmonotone equilibrium problems, stochastic and distributed equilibrium systems, bilevel equilibrium models, and fractional-order or time-delay dynamical systems. The development of adaptive and accelerated predefined-time schemes also remains an interesting direction for future research.

## Supporting information

S1 File MATLAB source codes used to reproduce all results presented in the manuscript. This ZIP file contains the MATLAB source codes (Fig1.m–Fig8.m) together with a README file describing the contents and execution procedure. Running these MATLAB scripts reproduces all figures, tables, and numerical results reported in the manuscript. No external datasets are required. The same materials are also publicly available in the Zenodo repository (https://doi.org/10.5281/zenodo.21293842). The contents of this file and the Zenodo repository are identical.
(ZIP)
