# Supplementary material for: Finite-time and predefined-time dynamical systems for solving strongly pseudomonotone mixed equilibrium problems
Source: PLoS One. 2026 Sep 1;21(9):e0357046. doi: 10.1371/journal.pone.0357046 (PMC13533400; doi:10.1371/journal.pone.0357046)
Supplement: Supplementary file 1 [file pone.0357046.s001.zip › MATLAB_Codes/README.pdf]

Title:
Finite-Time and Predefined-Time Dynamical Systems for Solving Strongly Pseudomonotone Mixed Equilibrium Problems

Manuscript ID: PONE-D-26-25567R1

Description
This folder contains the MATLAB source codes used to reproduce all figures, tables, and numerical results presented in the manuscript.

Running the **MATLAB scripts (Fig1.m--Fig8.m)** reproduces all figures, tables, and numerical results reported in the manuscript. **No external datasets are required** because all computations are performed directly by the provided MATLAB source codes.

The archive **MATLAB_Codes.zip** contains the following MATLAB files:

**MATLAB File**	**Description**
Fig1.m	Reproduces Fig. 1
Fig2.m	Reproduces Fig. 2
Fig3.m	Reproduces Fig. 3
Fig4.m	Reproduces Fig. 4
Fig5.m	Reproduces Fig. 5
Fig6.m	Reproduces Fig. 6
Fig7.m	Reproduces Fig. 7
Fig8.m	Reproduces Fig. 8

All MATLAB source codes were developed and tested using **MATLAB R2024a**.

These files are provided to facilitate the verification and reproducibility of the results reported in the manuscript.

The same materials are also publicly available in the Zenodo repository:
https://doi.org/10.5281/zenodo.21293842
